# Immunogenicity and safety of quadrivalent influenza vaccine among young and older adults in Tianjin, China: implication of immunosenescence as a risk factor

**DOI:** 10.1186/s12979-023-00364-6

**Published:** 2023-07-27

**Authors:** Tongling Xiao, Miaomiao Wei, Xiaokun Guo, Yu Zhang, Zhongyan Wang, Xiaoshuang Xia, Xuemei Qi, Lin Wang, Xin Li, Sean X. Leng

**Affiliations:** 1 Department of Neurology, The Second Hospital of Tianjin Medical University, 23 Pingjiang Road, Tianjin, 300211 China; 2 Department of Geriatrics, The Second Hospital of Tianjin Medical University, Tianjin, China; 3 Division of Geriatric Medicine and Gerontology, Department of Medicine, Johns Hopkins University School of Medicine, Baltimore, MD USA; 4 School of Medicine and Bloomberg School of Public Health, Division of Geriatric, Johns Hopkins Center On Aging and Immune Remodeling, Johns Hopkins University, JHAAC Room 1A.38A, 5501 Hopkins Bayview Circle, Baltimore, MD 21224 USA

**Keywords:** Quadrivalent inactivated influenza vaccine, Immunogenicity, Safety, Older adults, Aging, Immunosenescence

## Abstract

**Background:**

Older adults are more vulnerable to seasonal influenza than younger adults. The immune responses of older persons to the influenza vaccine are usually poorer than those of young individuals, which is hypothesized due to immunosenescence. We conducted a study to evaluate the immunogenicity and safety of a quadrivalent inactivated influenza vaccine (IIV4) in a total of 167 young (< 65 years, *n* = 79) and older (≥ 65 years, *n* = 88) adults from October 2021 to March 2022 in Tianjin, China. A single dose was administered to all participants. Blood samples were collected and strain-specific hemagglutination inhibition (HAI) antibody titers were measured before and 21 to 28 days after vaccination. Safety information was also collected for 28 days and 6 months after vaccination. Differences in immunogenicity and safety were compared between young and old age groups, and multivariate logistic regression was used to estimate the effect of age and other factors on HAI antibody responses.

**Results:**

Overall, geometric mean titers (GMTs) against all four vaccine strains in older adults were lower than those in the young, whereas the seroconversion rates (SCRs) were similar. Multivariate logistic regression analysis showed that age, influenza vaccination history, and pre-vaccination HAI titers were independent factors affecting SCRs and seroprotection rates (SCRs). Older age had significant negative impact on SCRs against H1N1 (OR, 0.971; 95% CI: 0.944–0.999; *P* = 0.042) and B/Victoria (OR, 0.964; 95% CI: 0.937–0.992; *P* = 0.011). In addition, there was a significant negative correlation between chronological age (years) and post-vaccination HAI titers against H1N1 (rho = -0.2298, *P* < 0.0001), B/Victoria (rho = -0.2235, *P* = 0.0037), and B/Yamagata (rho = -0.3689, *P* < 0.0001). All adverse events were mild (grade 1 or grade 2) that occurred within 28 days after vaccination, and no serious adverse event was observed.

**Conclusions:**

IIV4 is immunogenic and well-tolerated in young and older adults living in Tianjin, China. Our findings also indicate that age is an independent factor associated with poorer humoral immune responses to IIV4.

**Supplementary Information:**

The online version contains supplementary material available at 10.1186/s12979-023-00364-6.

## Background

Seasonal influenza is a common respiratory viral infection caused by the influenza viruses and represents a significant global health burden. Older adults and those with chronic diseases are more susceptible to severe influenza and its complications than young people [1] with higher case-fatality [2]. According to the World Health Organization (WHO), influenza causes 3–5 million severe cases and 290 000 to 650 000 influenza-associated respiratory deaths each year [2, 3], approximately 67% of which occur among people aged 65 and older [4]. To date, annual influenza vaccination remains a primary means of influenza prevention. Age-associated functional decline of the immune system, or “immunosenescence”, has been implicated as a key determinant making older adults not only more susceptible to infectious pathogens but also less responsive to vaccination [5, 6].

Influenza vaccines have been shown to reduce the risk of influenza infection and its adverse health outcomes in older adults [7, 8]. However, numerous studies have reported that influenza vaccine responses are typically diminished in older persons compared to their young counterparts, resulting in poorer antibody responses, lower seroconversion rates, and reduced efficacy and effectiveness [9–12]. Indeed, the rate of seroconversion as defined by fourfold or higher increase of HAI antibody titer after vaccination is much lower in older adults than young individuals [10]. The standard-dose influenza vaccine had a 70–90% of efficacy among young adults in preventing laboratory-confirmed influenza (LCI) but just 17–53% of efficacy in older adults [13, 14]. In a systemic review and meta-analysis, Rondy and colleagues have shown that influenza vaccination provides a 51% reduction in LCI related hospitalization in adults aged 18–65 years compared to a 37% reduction in those older than 65 years [15]. Taken together, these studies provide strong evidence supportive of impaired immune responses to influenza vaccines in older adults likely due to immunosenescence. Therefore, further research to better understand the impact of age and immunosenescence on antibody responses to influenza vaccination in older adults is indicated.

Currently, influenza vaccine coverage in China is only 2–3% and likely is even lower in older adults, except for some regions with supportive policies for vaccine cost reimbursement [16]. A previous survey by our group showed that influenza vaccination rate in Tianjin was about 1% in 2018, and the immunogenicity and safety of influenza vaccines for residents in Tianjin, China have not been evaluated. This study aimed to evaluate the immunogenicity and safety of a quadrivalent inactivated influenza vaccine (IIV4) among young and older adults living in Tianjin and determine whether there are age-specific differences.

## Results

### Immunogenicity

A total of 178 individuals were enrolled and 167 (93.8%) completed the study. Table 1 summarizes participants’ baseline demographic and clinical characteristics. Table 2 shows vaccine immunogenicity parameters as measured by hemagglutination inhibition (HAI) antibody response, including geometric mean titers (GMTs), GMT ratio, seroconversion rate (SCR), and pre- and post-vaccination seroprotection rate (SPR). The differences in all measurements between the two age groups were also evaluated (Table 2).

**Table 1:** Baseline characteristics of study participants (*N* = 167)

Characteristic	< 65 years (*N* = 79)	≥ 65 years (*N* = 88)	Total (*N* = 167)
Sex, n (%)			
Male	26 (32.9)	47 (53.4)	73 (43.7)
Age, years, n (%)			
Mean (SD)	47.3 (13.7)	70.9 (6.0)	59.7 (15.7)
Median (range)	53 (23–64)	69 (65–89)	65 (23–89)
Comorbid diseases, n (%)			
Hypertension	15 (19.0)	51 (58.0)	66 (39.5)
CHD	6 (7.6)	15 (17.0)	21 (12.6)
Diabetes	5 (6.3)	19 (21.6)	24 (14.4)
Hyperlipidemia	10 (12.7)	40 (45.5)	50 (29.9)
IV history	25 (31.6)	26 (29.5)	51 (30.5)

*Abbreviations*: *SD* Standard deviation, *CHD* Coronary heart disease, *IV history* Influenza vaccination history in the previous year

**Table 2:** Immunogenicity of IIV4 by young (< 65 years) and old (≥ 65 years) age groups

	GMT (95% CI) ^a^		GMT ratio (95% CI) ^a^	SCR (%, 95% CI) ^b^	SPR (%, 95% CI) ^b^	
	Pre-vaccination	Post-vaccination			Pre-vaccination	Post-vaccination
H1N1	17.81 (15.51–20.44)	91.74 (73.72–114.18)	5.15 (4.23–6.28)	53.89 (46.02–61.62)	25.75 (19.30–33.08)	76.05 (68.84–82.30)
< 65 years	20.00 (16.34–24.48)	131.91 (94.07–184.98)	6.60 (4.90–8.88)	60.76 (49.13–71.56)	25.32 (16.20–36.36)	81.01 (70.62–88.97)
≥ 65 years	16.04 (13.27–19.39)	66.22 (50.47–86.88)	4.13 (3.18–5.36)	47.73 (36.96–58.65)	26.14 (17.34–36.59)	71.59 (60.98–80.70)
*P*	0.117	0.002	0.018	0.092	0.904	0.154
H3N2	12.93 (11.35–14.74)	58.36 (50.29–67.72)	4.51 (3.77–5.4)	53.89 (46.02–61.62)	16.77 (11.44–23.31)	79.64 (72.73–85.47)
< 65 years	14.46 (11.93–17.52)	58.85 (47.13–73.48)	4.07 (3.14–5.28)	53.16 (41.60–64.49)	16.46 (9.06–26.49)	82.28 (72.06–89.96)
≥ 65 years	11.71 (9.79–14.00)	57.92 (47.23–71.03)	4.95 (3.84–6.38)	54.55 (43.58–65.20)	17.05 (9.87–26.55)	77.27 (67.11–85.53)
*P*	0.097	0.574	0.269	0.858	0.919	0.423
B/Victoria	15.27 (13.41–17.39)	76.43 (65–89.87)	5 (4.33–5.78)	66.47 (58.76–73.58)	20.36 (14.53–27.27)	83.23 (76.69–88.56)
< 65 years	17.08 (14.51–20.10)	87.34 (71.22–107.11)	5.11 (4.17–6.27)	72.15 (60.93–81.65)	17.72 (10.04–27.94)	89.87 (81.02–95.53)
≥ 65 years	13.81 (11.32–16.85)	67.80 (52.91–86.88)	4.91 (3.99–6.04)	61.36 (50.38–71.56)	22.73 (14.47–32.89)	77.27 (67.11–85.53)
*P*	0.065	0.05	0.515	0.14	0.423	0.03
B/Yamagata	30.8 (26.46–35.85)	147.25 (125.05–173.41)	4.78 (4.02–5.68)	64.07 (56.30–71.34)	62.87 (55.07–70.21)	94.61 (90.02–97.51)
< 65 years	44.44 (36.31–54.40)	187.37 (155.07–226.41)	4.22 (3.25–5.47)	56.96 (45.33–68.06)	77.22 (66.40–85.90)	100.00 (95.44–100.00)
≥ 65 years	22.16 (18.08–27.16)	118.61 (91.95–153.01)	5.35 (4.25–6.75)	70.45 (59.78–79.71)	50.00 (39.15–60.85)	89.77 (81.47–95.22)
*P*	< 0.001	0.012	0.131	0.07	< 0.001	0.003

*Abbreviations*: *CI* Confidence interval, *GMT* Geometric mean titer, *GMT ratio* Geometric mean titer ratio, *SCR* Seroconversion rate, *SPR* Seroprotection rate

^a^ Comparisons of GMT and GMT ratio between the two age groups were performed by Mann–Whitney U test as appropriate

^b^ Comparisons of SCR and SPR between the two age groups were performed by Chi-square or Fisher's exact test as appropriate

**Fig. 1:**
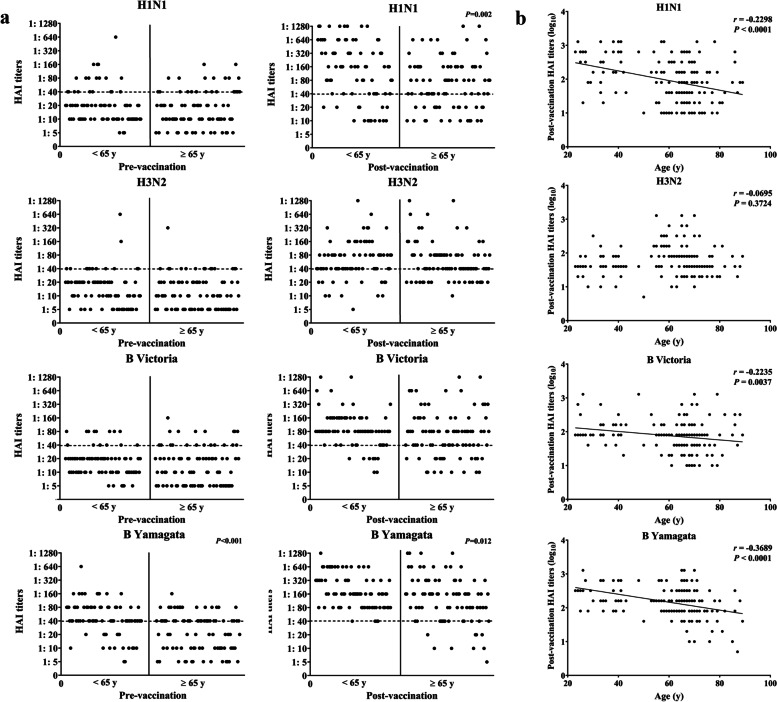
Immunogenicity of IIV4 among young and older adults living in Tianjin, China. **a** Dotplots depicting pre- and post-vaccination strain-specific HAI antibody titers as indicated with each panel representing one vaccine strain. Data points for the young (< 65 y) and old age (≥ 65 y) are separated by a vertical line and seroprotective titer 1:40 is illustrated by a horizonal dotted line. P values of statistical significance between the young and old age groups when observed are also shown. **b** Dotplots illustrating relationship between chronological age and post-vaccination HAI titers with each panel representing one vaccine strain. The HAI titers were log_10_ transformed, and the correlation was determined using Spearman's rank correlation test. Significant correlation when observed is indicated by a regression line with *r* and *P* value shown at the right upper corner of the corresponding panel

Overall, both pre- and post- vaccination GMTs against all four vaccine strains in the older adult group (≥ 65 years) were lower than those in the young group (< 65 years). For example, pre-vaccination GMT against the B/Yamagata strain was significantly higher in the young group than in the old group (*P* < 0.001). Except for H3N2, post-vaccination GMTs were significantly higher in the young group than in the old group (*P* < 0.05) (Fig. 1a). GMTs against all four vaccine strains increased by 4.07 to 6.60-fold in both age groups after vaccination. Among them, the GMT against H1N1 increased to a greater extent in the < 65 years age group than in the ≥ 65 years age group (6.60-fold versus 4.13-fold; *P* = 0.018). Spearman's rank correlation test was performed to evaluate the relationship between (continuous variable as counted by years) and post-vaccination HAI titers. There was a significant negative correlation between age and post-vaccination HAI titers (log_10_ transformed) against H1N1 (rho = -0.2298, *P* < 0.0001), B/Victoria (rho = -0.2235, *P* = 0.0037), and B/Yamagata (rho = -0.3689, *P* < 0.0001) as shown in Fig. 1b. No such correlation was observed between age and post-vaccination HAI titers against H3N2.

**Fig. 2:**
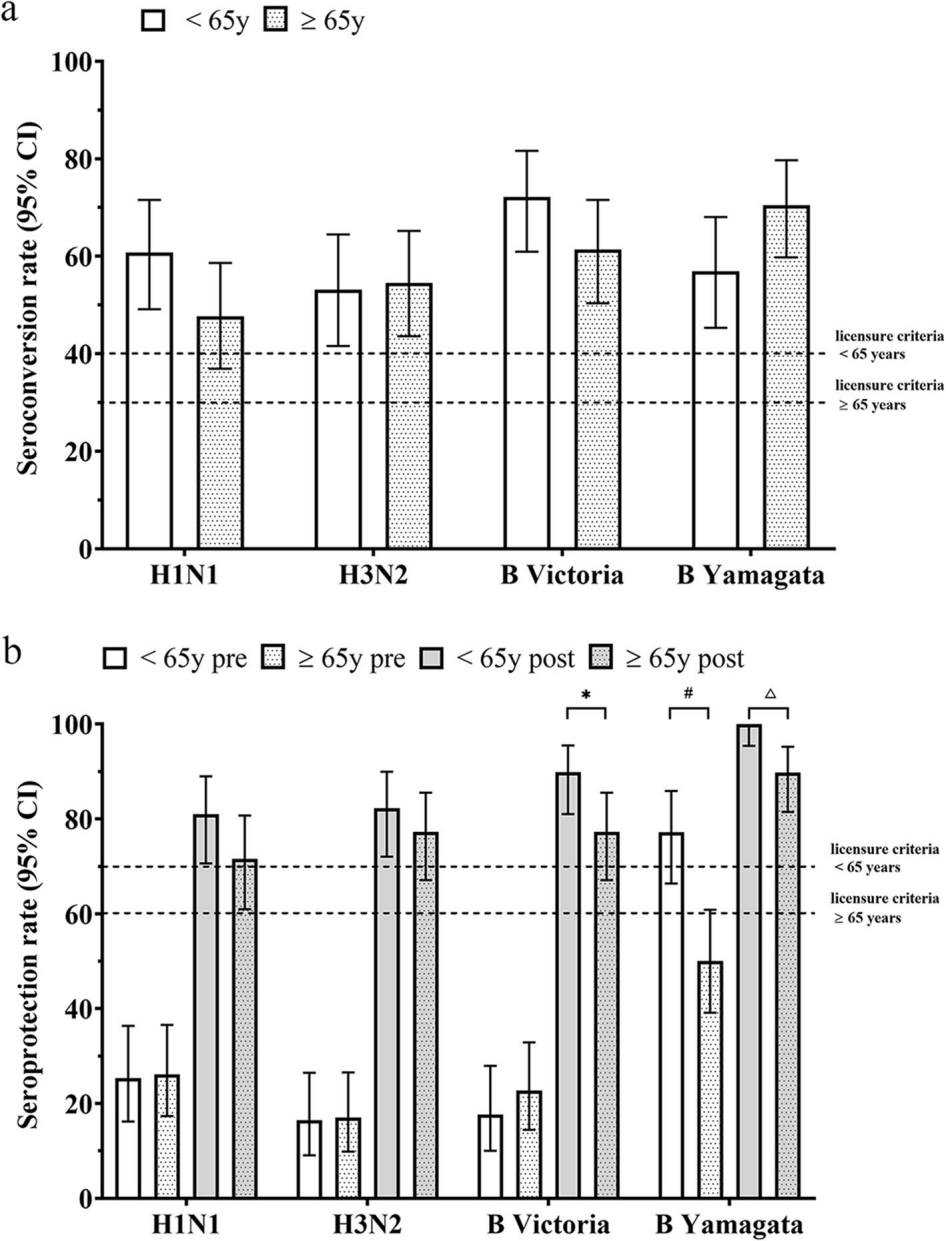
Comparison of SCRs and SPRs by age groups. **a** Bar graphs illustrating seroconversion rates (SCRs) against each of the four vaccine strains, young *versus* old age groups as indicated. CBER licensure criteria are indicated by two horizonal dotted lines. **b** Bar graphs illustrating seroprotection rate (SPRs) against each of the four strains, young *versus* old age groups as indicated. CBER licensure criteria are indicated by two horizonal dotted lines. Open bars, pre-vaccination SPRs; Gray colored bars, post-vaccination SPRs. ^*^P < 0.05 post-vaccination SPR against B/Victoria between the young and old age groups; ^#^*P* < 0.001 pre-vaccination SPR against B/Yamagata between the young and old age groups; ^△^*P* < 0.01 post-vaccination SPR against B/Yamagata between the young and old age groups

Pre-vaccination SPRs of each age group were 16.46% to 77.22%. In addition, post-vaccination SPRs against influenza B strains were markedly lower in the ≥ 65 years age group than in the < 65 years age group (89.87% vs. 77.27% against B/Victoria, *P* = 0.03; 100% vs. 89.77% against B/Yamagata, *P* = 0.003) (Table 2). Overall, SCRs against H1N1, H3N2, B/Victoria and B/Yamagata strains were 53.89%, 53.89%, 66.47%, and 64.07%, respectively. SCRs against each of the four vaccine strains were similar between the two age groups, ranging from 47.73% to 72.15%. Interestingly, SCR against B/Yamagata in the older adult group was higher than that in the young group, which might be attributed to the high pre-vaccination HAI antibody titers in the young group (Table 2). Taken together, these results indicate that SCRs and SPRs differed at various degrees between the two age groups, but the lower bounds of the two-sided 95% confidence interval (CI) of SCRs and SPRs against all four vaccine strains in both age groups met the immunogenicity criteria [17] set by the Center for Biologics Evaluation and Research (CBER) (Fig. 2a and b).

### Factors associated with seroconversion and seroprotection

Multivariate logistic regression analyses revealed that age, influenza vaccination history, and pre-vaccination HAI antibody titer were independent factors affecting SCRs and SPRs against all four vaccine strains, adjusting for male sex, number of days after vaccine administration for blood sample collection, and common comorbid conditions including hypertension, hyperlipidemia, coronary heart disease, and diabetes mellitus (Table 3). Specifically, older age negatively impacted on SCRs against H1N1 (OR, 0.971; 95% CI: 0.944–0.999) and B/Victoria (OR, 0.964; 95% CI: 0.937–0.992), as well as on post-vaccination SPRs against B/Victoria (OR, 0.948; 95% CI: 0.901–0.997). The association between older age and post-vaccination SPR against B/Yamagata approached statistical significance (OR, 0.742; 95% CI: 0.550–1.001; *P* = 0.051). Influenza vaccination in the previous year was positively associated with pre-vaccination SPR against H1N1 (OR, 7.794; 95% CI: 3.452–17.595) and B/Yamagata (OR, 5.868; 95% CI: 2.357–14.610), whereas it was negatively associated with SCRs against H1N1 (OR, 0.153; 95% CI: 0.064–0.366) and B/Yamagata (OR, 0.381; 95% CI: 0.170–0.855). The effects of other variables including male sex, number of days after vaccine administration for blood sample collection, and common comorbid conditions (hypertension, hyperlipidemia, coronary heart disease, and diabetes mellitus) were not statistically significant (Supplemental Table S1).

**Table 3:** Multivariate logistic regression analyses for SCR and SPR

	SCR		Pre-vaccination SPR		Post-vaccination SPR	
	aOR (95% CI)	*P*	aOR (95% CI)	*P*	aOR (95% CI)	*P*
H1N1						
Age, years	0.971 (0.944–0.999)	0.042	0.996 (0.965–1.027)	0.794	0.964 (0.923–1.007)	0.096
IV history	0.153 (0.064–0.366)	< 0.001	7.794 (3.452–17.595)	< 0.001	0.357 (0.114–1.112)	0.076
Pre-vaccination HAI	0.997 (0.988–1.007)	0.598	——	——	1.184 (1.089–1.287)	< 0.001
H3N2						
Age, years	0.998 (0.971–1.027)	0.908	1.007 (0.974–1.040)	0.693	1.019 (0.985–1.053)	0.274
IV history	0.691 (0.312–1.533)	0.363	1.655 (0.694–3.946)	0.256	0.574 (0.231–1.425)	0.231
Pre-vaccination HAI	0.911 (0.877–0.946)	< 0.001	——	——	1.063 (1.014–1.115)	0.012
B/Victoria						
Age, years	0.964 (0.937–0.992)	0.011	1.017 (0.986–1.050)	0.286	0.948 (0.901–0.997)	0.038
IV history	0.919 (0.413–2.044)	0.835	2.21 (0.980–4.985)	0.056	0.329 (0.092–1.170)	0.086
Pre-vaccination HAI	0.973 (0.958–0.989)	0.001	——	——	1.153 (1.052–1.264)	0.002
B/Yamagata						
Age, years	0.985 (0.956–1.016)	0.343	0.93 (0.898–0.963)	< 0.001	0.742 (0.550–1.001)	0.051
IV history	0.381 (0.170–0.855)	0.019	5.868 (2.357–14.610)	< 0.001	0.013 (0.000–10.369)	0.203
Pre-vaccination HAI	0.983 (0.972–0.994)	0.003	——	——	1.27 (0.981–1.643)	0.069

*Abbreviations*: *SCR* Seroconversion rate, *SPR* Seroprotection rate, *CI* Confidence interval, *aOR* Adjusted odds ratio (adjusted for male sex, number of days after vaccine administration for blood sample collection, and common comorbid conditions including hypertension, hyperlipidemia, coronary heart disease, and diabetes mellitus), *IV history* influenza vaccination history in the previous year, *HAI* Hemagglutination inhibition antibody titer

We further performed logistic regression analyses among participants with a pre-vaccination HAI antibody titer < 40 and those with a pre-vaccination HAI antibody titer ≥ 40, adjusting for male sex, number of days after vaccine administration for blood sample collection, and common comorbid conditions including hypertension, hyperlipidemia, and diabetes mellitus. Coronary heart disease was not included in these subgroup analyses due to too few cases. In the former, the results were similar to those for the entire study population. Older age had negative impact on SCRs against H1N1 (OR, 0.955; 95% CI: 0.92–0.992), B/Victoria (OR, 0.949; 95% CI: 0.911–0.988), and B/Yamagata (OR, 0.764; 95% CI: 0.629–0.929) (Table 4). In the latter, all participants had HAI antibody titers ≥ 40 at both pre- and post-vaccination time points and SCRs were low. There was no significant association between age and SCR against any of the four vaccine strains in this group (Table 5). More details of these subgroup analyses can be found in Supplement Table S2.

**Table 4:** Multivariate logistic regression analyses for SCR and post-vaccination SPR in participants with pre-vaccination HAI antibody titer < 40

	SCR		Post-vaccination SPR	
	aOR (95% CI)	*P*	aOR (95% CI)	*P*
H1N1 (*N* = 124)				
Age, years	0.955(0.92–0.992)	0.018	0.963(0.922–1.006)	0.091
IV history	0.341(0.122–0.952)	0.04	0.362(0.118–1.114)	0.076
Post-vaccination HAI	1.028(0.955–1.106)	0.461	1.165(1.062–1.277)	0.001
H3N2 (*N* = 139)				
Age, years	1.006(0.975–1.037)	0.722	1.023(0.987–1.06)	0.218
IV history	0.537(0.235–1.228)	0.141	0.461(0.175–1.21)	0.116
Post-vaccination HAI	0.933(0.878–0.992)	0.027	1.099(1.016–1.189)	0.018
B/Victoria (*N* = 133)				
Age, years	0.949(0.911–0.988)	0.011	0.947(0.901–0.996)	0.035
IV history	0.461(0.164–1.291)	0.14	0.348(0.099–1.224)	0.1
Post-vaccination HAI	1.044(0.967–1.127)	0.275	1.148(1.038–1.27)	0.007
B/Yamagata (*N* = 62)				
Age, years	0.764(0.629–0.929)	0.007	0.752(0.573–0.988)	0.041
IV history	0.655(0.034–12.529)	0.779	0.015(0.000–11.705)	0.217
Post-vaccination HAI	0.915(0.76–1.103)	0.352	1.255(0.931–1.691)	0.136

*Abbreviations*: *N* Number of subjects in group, *CI* Confidence interval, *aOR* Adjusted odds ratio (adjusted for male sex, number of days after vaccine administration for blood sample collection, and common comorbid conditions including hypertension, hyperlipidemia, and diabetes mellitus); *IV history* influenza vaccination history in the previous year, *HAI* hemagglutination inhibition antibody titer

**Table 5:** Multivariate logistic regression analyses for SCR in participants with pre-vaccination HAI antibody titer ≥ 40

	SCR	
	aOR (95% CI)	*P*
H1N1 (*N* = 43)		
Age, years	1.018(0.945–1.096)	0.634
IV history	0.006(0.000–0.151)	0.002
Pre-vaccination HAI	0.997(0.987–1.008)	0.662
H3N2 (*N* = 28)		
Age, years	——	0.889
IV history	——	0.89
Pre-vaccination HAI	——	0.973
B/Victoria (*N* = 34)		
Age, years	0.993(0.921–1.071)	0.862
IV history	2.486(0.323–19.153)	0.382
Pre-vaccination HAI	0.976(0.94–1.014)	0.212
B/Yamagata (*N* = 105)		
Age, years	1.006(0.971–1.041)	0.752
IV history	0.339(0.129–0.887)	0.028
Pre-vaccination HAI	0.99(0.978–1.003)	0.126

*Abbreviations*: *N* Number of subjects in group, *CI* Confidence interval, *aOR* Adjusted odds ratio (adjusted for male sex, number of days after vaccine administration for blood sample collection, and common comorbid conditions including hypertension, hyperlipidemia, and diabetes mellitus), *IV history* Influenza vaccination history in the previous year, *HAI* Hemagglutination inhibition antibody titer

### Safety

Overall, 32 participants (19.16%) experienced 51 adverse events (AEs) within 28 days after vaccination (Table 6), most of which were solicited AEs. Among these 32 participants, 19 (11.38%) experienced local AEs, and the most common one was pain at vaccine injection site. Systemic AEs occurred in 17 participants (10.18%), and the most frequent one was fever. According to the guidelines [18] issued by the China National Medical Products Administration (NMPA), all AEs observed in this study were grade 1 or grade 2, and no serious adverse events or medically attended event occurred within six months.

**Table 6:** AEs in young and older adult groups

AE, n (%)	< 65 years (*n* = 79)	≥ 65 years (*n* = 88)	*P*
Participants with AEs	18 (22.78)	14 (15.91)	0.260
Participants with local AE	11 (13.92)	8 (9.09)	0.326
Participants with systemic AE	10 (12.66)	7 (7.95)	0.316
Total AE	30(40.0)	21(23.9)	
Immediate unsolicited AE	0 (0.00)	1 (1.14)	
Unsolicited AE	2 (2.53)	0 (0.00)	
Solicited AE	28(35.4)	20(22.7)	
Local AE	15(19.0)	11(12.5)	
Redness	3 (3.80)	2 (2.27)	
Swelling	1 (1.27)	2 (2.27)	
Pain	8 (10.13)	5 (5.68)	
Rash	1 (1.27)	0 (0.00)	
Itch	2 (2.53)	1 (1.14)	
Induration	0 (0.00)	1 (1.14)	
Systemic AE	15(19.0)	10(11.4)	
Fever	5 (6.33)	4 (4.55)	
Myalgia	4 (5.06)	1 (1.14)	
Fatigue	3 (3.80)	3 (3.41)	
Headache	2 (2.53)	1 (1.14)	
Cough	0 (0.00)	1 (1.14)	
Nausea & Vomiting	1 (1.27)	0 (0.00)	
SAE	0 (0.00)	0 (0.00)	
MAE	0 (0.00)	0 (0.00)	

*Abbreviations*: *AE* Adverse event, *SAE* Serious adverse event, *MAE* Medically attended event. Comparisons of AEs between the two age groups were performed by Chi-square test

In addition, we compared AE rates between the two age groups. As shown in Table 6, the AE rate in the < 65 age group was slightly higher than that in the ≥ 65 age group for both local and systemic AEs, but the difference was not statistically significant.

## Discussion

The results of this study demonstrate that IIV4 was immunogenic and well tolerated among young and older adults living in Tianjin, China. Strain-specific HAI antibody titers increased after vaccination and the SCRs and SPRs met the CBER criteria in all participants. However, both pre- and post- vaccination GMTs against the majority of the vaccine strains were significantly lower in the older adults than those in the young. Multivariate analyses indicate that age, prior influenza vaccination history, and pre-vaccination HAI antibody titer were independent factors with significant impact on seroconversion and seroprotection. In addition, there was a low incidence of AEs (19.16%) among the participants, with no serious AEs observed.

A recent systematic review on the immunogenicity of IIV4 in young and older adults from different countries reported that pooled SCRs against H1N1, H3N2, B/Victoria, and B/Yamagata strains were 65%, 65%, 63%, and 63%, respectively [19]. The SCRs against influenza vaccine B strains were similar to those in our study, while the SCRs against influenza vaccine A strains were slightly higher than ours, which might in part be attributed to the fact that more participants enrolled in our study were older adults. In our study, SCRs against H1N1, H3N2, B/Victoria, and B/Yamagata were 47.73%, 54.55%, 61.36%, and 70.45% in the older adult group, and 60.76%, 53.16%, 72.15%, and 56.96% in the young group, respectively. In a phase III trial in South Korea, the corresponding SCRs were 42.2%, 50.0%, 35.9%, and 46.9% in the older adults (≥ 65), 57.7%, 60.4%, 52.9%, and 53.7% in the young (< 65), which were lower than those in our study [20]. Some studies in the United States and European countries also had lower SCRs in older adults than ours [21, 22]. However, influenza vaccination coverage in older adults is very high in these countries, leading to high pre-vaccination HAI antibody titers which can impact SCRs [12].

Consistent with previous studies [23–25], results from our comparative analyses of GMT ratios and SCRs between young and old age groups demonstrate that older adults had less robust HAI antibody responses to IIV4 than the young. However, few studies have explored the independent influence of age as a risk factor for poor antibody responses in older adults. A study conducted in Hong Kong among older adults vaccinated with IIV4 in 2003 showed that age was not an independent predictor of poor immunogenicity [26]. This might be attributable to the limited age distribution of the study population as all participants in that study were over 60 years of age, and the author only analyzed age as a categorical variable. Of note, there was an independent association between sex and SCR against H3N2 in that study, i.e., SCR against H3N2 was higher in women (OR, 4.84; 95% CI: 1.31–17.91; *P* = 0.018), and this is consistent with our results. Another study explored the immunogenicity of IIV4 among vaccinated individuals in Shenzhen and Changzhou. In that study, multivariate logistic regression analyses revealed an independent influence of age on SCRs against H1N1 (OR, 5.515; 95% CI: 1.888–16.109; *P* = 0.002), B/Victoria (OR, 3.755; 95% CI: 1.305–10.800; P = 0.014), and B/Yamagata (OR, 5.775; 95% CI: 1.938–17.208; *P* = 0.002) [24]. While such sex differences in humoral immune responses to influenza vaccines are hypothesized to be caused by the impact of sex hormones on the immune system [27], a more recent study showed sex-specific effects of aging on humoral immune responses to repeated vaccination with the high-dose IIV3 among older adults 75 years and older with women aged many years after menopause [12], arguing against this hypothesis. In our study, age was independently associated with low SCRs against only two influenza virus strains, i.e., H1N1 and B/Victoria. The biological mechanisms underlying this association remains unclear and requires further investigations.

A Cochrane systemic review and meta-analysis demonstrate that influenza vaccine effectiveness is variable among different seasons and against different circulating influenza virus strains with estimated vaccine effectiveness against medically attended influenza ranging from 57 to 68% in young adults vs. 10% to 49% in older adults [7, 28]. The reduced influenza vaccine effectiveness in older adults is considered due to immunosenescence, which is an age-associated immunodeficient state characterized by thymic involution and functional decline, reduced T-cell proliferation, and impairment of humoral and cellular immunity [5]. Older adults manifest an overall decline in immune function, leading to increased susceptibility to infectious diseases and severity, poor immune response to vaccines, and increased incidence of cancer and autoimmune diseases [29].

The mechanism for immunosenescence contributing to poor vaccine responses in older adults is likely multifaceted, involving declines in both innate [30] and adaptive immunity [31, 32]. For innate immunity, one hypothesis is that immunosenescence leads to dysregulation of toll-like receptor (TLR) signaling pathways and cytokine production by macrophages [33, 34]. Previous studies have found that a decline in influenza-induced production of interferon (IFN)-α in older adults is associated with defective TLR signaling, specifically TLR7 [35]. Alterations in the function of plasmacytoid dendritic cells [36, 37] and phenotypic transition of natural killer cells [38, 39] may also be detrimental factors impacting on vaccine-induced immune response in older adults. The role of immunosenescence in the adaptive immunity has been a focus of intense research. As the thymus involutes, naïve T cells decline and immune repertoire is skewed to memory phenotype [32, 40, 41]. Meanwhile, Accumulation of intrinsic defects in effector T cells [42], imbalance of cytokine production by helper T cells [43], and diminished responses of memory T cells to antigen stimulation [44] are all associated with a weakened immune response in older adults upon influenza infection or vaccination. Moreover, changes in the levels of switched memory B cells [45, 46], as well as B cell receptor diversity [47, 48] are also crucial factors influencing humoral immune responses to vaccination in older adults. Comprehensive and in-depth investigations employing cutting edge technologies including cellular indexing of transcriptomes and epitopes by sequencing (CITE-seq) and single cell RNA-seq to further elucidate the underlying mechanisms are indicated.

In addition to age, prior influenza vaccination history also affects SCRs and pre- and post-vaccination SPRs. In this study, 30.54% participants had been vaccinated against influenza in the previous year. Studies have shown that prior influenza vaccination is associated with lower antibody responses to subsequent influenza vaccination [49, 50], which is also the case in our study, especially for H1N1 and B/Yamagata, Influenza vaccination within the prior year was an independent factor associated with reduced SCRs.

In terms of safety, IIV4 was well tolerated in our study, and no AE of grade 3 or above was observed. Consistent with previous studies, our results demonstrate that AEs were slightly more frequent in young participants than those in older participants [51, 52]. Age-related functional decline of cells that participate in local and systemic inflammatory response may in part explain this phenomenon [53].

This study has several limitations. First, the study sample size was small and we did not include unvaccinated individuals as a control group. Secondly, no additional time points were included to further monitor changes in HAI antibody titers over time. As such, the study does not provide insights into the durability of the vaccine-induced humoral immune response. Finally, vaccine immunogenicity as measured by strain-specific HAI antibody titers does not necessarily represent clinical protection. Despite these limitations, our study is the first to evaluate the immunogenicity and safety of IIV4 in an adult population in Tianjin, China. The results indicate that age can independently affect humoral immune response to influenza vaccines.

## Conclusions

A quadrivalent inactivated influenza vaccine (IIV4) was immunogenic and safe in immunizing the adult population in Tianjin, China. Age was an independent risk factor for impaired humoral immune responses to IIV4.

## Methods

### The study population

This study was conducted among adults aged 18–64 years and older adults ≥ 65 years in Tianjin, China. All participants were community-dwelling recruited from the Physical Examination Center of the Second Hospital of Tianjin Medical University between October 2021 and March 2022. Exclusion criteria were as follows: (1) individuals with confirmed influenza infection or those who received the influenza vaccine within 6 months before the study; (2) allergy to eggs or any components of the vaccine; (3) had a previous severe adverse reaction to any vaccination; (4) immune-related disorders including Guillain–Barre syndrome; (5) individuals received immunosuppressive therapy or systemic steroids in the last 6 months; (6) individuals treated with immunoglobulin or blood products in the past 3 months; (7) bleeding disorders or other conditions that might lead to severe bleeding; (8) uncontrolled severe chronic diseases (cardiovascular and cerebrovascular diseases, respiratory diseases, hepatic and renal insufficiency, chronic infection, etc.); and (9) a history of developmental delay, psychological disease, or epilepsy.

A total of 178 participants were enrolled in this study; 167 participants received vaccination and completed the study with 11 dropouts. Baseline clinical characteristics of the participants are shown in Table 1. There were more female participants (56.29%) than males (43.71%). The overall age distribution ranged from 23 to 89 years, with a median age of 65. Specifically, 79 (47.31%) participants were under 65 years old and 88 (52.69%) participants were aged 65 years or older. Among all participants, 51 (30.54%) had received one dose of influenza vaccine in the previous year (2020–2021), and 26 (51%) were in the older adult group and 25 (49%) in the young group (Table 1).

### Collection of data and blood samples

Participants were recruited by trained medical staff with informed consent. Information of participants’ demographic and clinical characteristics, including name, age, sex, and medical history was obtained and recorded by in-person interviews. Venous blood samples were collected for serological analysis at baseline (day 0, before vaccination) and 21–28 days after vaccination. After centrifugation, 1 ml of serum from each sample was transported to the Tianjin Center for Disease Control and stored at –80 °C.

### Influenza vaccination

The vaccine administered in this study was a quadrivalent inactivated split-virion influenza vaccine (IIV4) produced in embryonated chicken eggs and was approved for use by the National Institutes for Food and Drug Control of China (Hualan Biological Engineering, China Drug Approval No.: S20083016). Each dose of IIV4 contained 60 μg (15 μg of each strain) of hemagglutinin antigen (HA) from four influenza strains predicted by the WHO for the 2021–2022 influenza season in the Northern Hemisphere: A/Victoria/2570/2019 (H1N1) pdm09-like virus, A/Cambodia/e0826360/2020 (H3N2)-like virus, B/Washington/02/2019 (B/Victoria lineage)-like virus, and B/Phuket/3073/2013 (B/Yamagata lineage)-like virus. The vaccine was administered by intramuscular injection to the deltoid muscle.

### Immunogenicity assessments

Strain-specific hemagglutination inhibition (HAI) antibody titers were measured using standard hemagglutination inhibition assays, which were performed following the Standard Operating Procedures published by the Chinese Center for Disease Control and Prevention. Briefly, serum samples were pretreated with receptor-destroying enzyme (RDE) (Denka Seiken, Tokyo, Japan) at a 1:3 dilution ratio to inactivate nonspecific inhibitors. Turkey red blood cells (RBCs) were then added at a 1:20 dilution to remove non‐specific agglutinins. Starting with a 1:10 dilution, 25 μl of serially-diluted serum samples were mixed with 25 μl of four HA units of antigens on 96-well V-bottom microtiter plates. Fifty microliters of 1% turkey RBCs was added to each well and incubated for 30–60 min at room temperature. The plate was observed for hemagglutination. HAI titers were defined as the highest serum dilution that completely inhibited hemagglutination.

The following metrics were used for immunogenicity evaluation: geometric mean titer (GMT) was defined as the anti-log of the arithmetic mean of the log-transformed inverse titers (a titer of < 1:10 was calculated as 1:5); GMT ratio was obtained by computing the geometric mean of the log-transformed ratio of inverse titers before and after vaccination; seroconversion rate (SCR) was defined as the proportion of participants with an antibody titer of < 1:10 before vaccination and a titer of ≥ 1:40 after vaccination or a titer ≥ 1:10 before vaccination and a ≥ fourfold increase in titer after vaccination; and seroprotection rate (SPR) was defined as the proportion of participants with an antibody titer of ≥ 1:40. According to the "Guidance for Industry: Clinical Data Needed to Support the Licensure of Seasonal Inactivated Influenza Vaccines" issued by the Center for Biologics Evaluation and Research (CBER) and Food and Drug Administration (FDA) in 2007 [17]:

For adults < 65 years of age and for the pediatric population:

A) The lower bound of the two-sided 95% confidence intervals (CI) for SCR should meet or exceed 40%.
B) The lower bound of the two-sided 95% CI for SPR should meet or exceed 70%.

For adults ≥ 65 years of age:

A) The lower bound of the two-sided 95% CI for SCR should meet or exceed 30%.
B) The lower bound of the two-sided 95% CI for SPR should meet or exceed 60%.

### Vaccine safety assessment

All participants were immediately observed for at least 30 min after vaccination for safety and to monitor for immediate adverse events (AEs). Furthermore, participants or their families were asked to record solicited AEs from day 0–7 in diary cards, which were reviewed by medical staff at the time of the second blood collection. The unsolicited AEs were also reported by participants automatically up to 28 days by telephone after vaccination. Data on serious adverse events (SAEs) and medically attended events (MAEs) were collected 6 months after vaccination. According to "Guidelines for grading adverse events in clinical trials of vaccines for prophylaxis" issued by the China National Medical Products Administration (NMPA) [18], the severity of local and systemic AEs was categorized into four grades (grade 1, 2, 3, and 4).

### Statistical analysis

The two-sided 95% CI of SCR and SPR were calculated using Clopper-Pearson method. GMT, GMT ratio, and their 95% CIs in two age groups were calculated and compared after logarithmic transformation. Spearman's rank correlation test was used to assess the relationship between chronological age (continuous variable as counted by years) and post-vaccination HAI titers (log_10_ transformed). To investigate the independent association of age with SPR (both pre-vaccination and post-vaccination) and SCR, potentially important variables, including male sex, influenza vaccination history in the previous year, pre-vaccination HAI antibody titer, the number of days after vaccine administration for blood sample collection, and common comorbid conditions (hypertension, hyperlipidemia, coronary heart disease, and diabetes mellitus) were included in a multivariate logistic regression model. Sample size was estimated based on the effect-size described in a previous study with a similar study design to detect the difference in immunogenicity (Specifically SCR) of IIV4 between young (< 65) and older adults (≥ 65) [54]. Assuming 90% power and type I error α = 0.05 (two-sided), a sample size of 81 per group was needed for H1N, 52 for H3N2, 41 for B/Victoria, and 51 for B/Yamagata. PASS 11 was used for this sample size calculation [55].

Group differences were analyzed using two-sided t-test or Mann–Whitney U test for continuous variables and Chi-square or Fisher's exact test for categorical variables as appropriate. All statistical analyses were performed using IBM SPSS Statistics 26.0, and statistical significance was set at *P* < 0.05.

## Supplementary Information

**Additional file 1.**

**Additional file 2.**

## Data Availability

The datasets used in this study are available from the corresponding author upon reasonable request.

## Glossary

Adverse event: An adverse event (AE) is any untoward medical occurrence in a clinical study participant, temporally associated with the use of study intervention, whether or not considered related to the study intervention.
Geometric mean titer (GMT): The GMT is the anti-log of the arithmetic mean of the log-transformed inverse HAI antibody titers.
Hemagglutination inhibition antibody titer: The HAI/HI antibody titer is the inverse of the last dilution of serum that completely inhibited hemagglutination in a hemagglutination assay.
Immediate adverse event: Immediate adverse events are recorded to capture medically relevant unsolicited AEs which occur within the first 30 minutes after vaccination.
Immunogenicity: Immunogenicity is the ability of a foreign substance, such as an antigen, to provoke an immune response in the body of a human or other animal.
Local adverse event: An injection/administration site adverse event is an AE at and around the injection/administration site of the vaccine. Local AEs are commonly inflammatory reactions.
GMT ratio: The GMT ratio, also called GMT mean fold increase (MFI) or mean fold rise (MFR), is the geometric mean of the log-transformed ratio of inverse HAI antibody titers before and after vaccination.
Medically attended event: A Medically attended event (MAE) is a new onset or a worsening of a condition that prompts the participant or participant’s parent/legally acceptable representative to seek unplanned medical advice at a physician’s office or Emergency Department.
Serious adverse event: Any AE that results in death, is life-threatening, requires inpatient hospitalisation or prolongation of existing hospitalisation, results in persistent or significant disability/incapacity, or is another medically important event (not meet any of the above seriousness criteria, but which are considered as serious based on investigator medical judgment).
Seroconversion: Seroconversion refers the production of specific antibodies against specific antigens in the blood serum as a result of infection or immunization, including vaccination. Seroconversion for influenza vaccine was defined as an antibody titer of < 1:10 before vaccination and a titer of ≥ 1:40 after vaccination or a titer ≥ 1:10 before vaccination and a ≥ 4-fold increase in antibody titer after vaccination.
Seroprotection: Seroprotection refers the antibody titers against the specific antigens in the blood serum reach a serological immune protective level. Seroprotection for influenza vaccine was defined as an antibody titer of ≥ 1:40.
Solicited adverse event: A solicited adverse event is an “expected” adverse reaction (sign or symptom) observed and reported under the conditions (nature and onset) pre-listed in the protocol and Case report form.
Systemic adverse event: Systemic AEs are all AEs that are not injection or administration site AEs. They therefore include systemic manifestations such as headache, fever, as well as localized or topical manifestations that are not associated with the injection or administration site.
Unsolicited adverse event: An unsolicited AE is an observed AE that does not fulfill the conditions of solicited reactions, i.e., pre-listed in the Case report form in terms of diagnosis and onset window post-vaccination.
